# Epithelial–Mesenchymal Transition in the Resistance to Somatostatin Receptor Ligands in Acromegaly

**DOI:** 10.3389/fendo.2021.646210

**Published:** 2021-03-15

**Authors:** Joan Gil, Mireia Jordà, Berta Soldevila, Manel Puig-Domingo

**Affiliations:** ^1^ Endocrine Tumours Lab, Program of Predictive and Personalized Medicine of Cancer (PMPPC), Germans Trias i Pujol Research Institute (IGTP), Badalona, Spain; ^2^ Department of Endocrinology and Nutrition, Germans Trias i Pujol University Hospital, Badalona, Spain; ^3^ Department of Medicine, Autonomous University of Barcelona, Bellaterra, Spain

**Keywords:** epithelial–menchymal transition, somatostatin analogs, pituitary, E-cadherin, somatotroph adenoma, growth hormone, PitNETs, endocrine tumors

## Abstract

Epithelial-mesenchymal transition (EMT) is a dynamic process by which epithelial cells loss their phenotype and acquire mesenchymal traits, including increased migratory and invasive capacities. EMT is involved in physiological processes, such as embryogenesis and wound healing, and in pathological processes such as cancer, playing a pivotal role in tumor progression and metastasis. Pituitary tumors, although typically benign, can be locally invasive. Different studies have shown the association of EMT with increased tumor size and invasion in pituitary tumors, and in particular with a poor response to Somatostatin Receptor Ligands (SRLs) treatment in GH-producing pituitary tumors, the main cause of acromegaly. This review will summarize the current knowledge regarding EMT and SRLs resistance in acromegaly and, based on this relation, will suggest new biomarkers and possible therapies to SRLs resistant tumors.

## Introduction

Epithelial–mesenchymal transition (EMT) is a dynamic process that reorganizes the cell from an epithelial to a mesenchymal phenotype leading to functional changes in cell invasion and migration capacities (1). This process is triggered by microenvironment signals that cells receive which produce changes in gene expression and post-translational regulation mechanisms leading to the loss of epithelial characteristics (cell polarity, stable epithelial cell-cell junctions and interactions with extracellular matrix) and the acquisition of mesenchymal features (fibroblast-like morphology and increased migratory and invasive properties). Although it has been considered as a binary process for many years, EMT has been recently shown to occur through distinct transition cellular states that are driven by a network of transcription factors (EMT-TFs) (2, 3). *SNAI1-2*, *TWIST*, and ZEB protein families have been the most extensively studied EMT-TFs as they regulate the classical EMT focused on the repression of E-cadherin, the prototypic adhesion molecule; however, the list of EMT-TFs has largely grown in the last years (4).

EMT was first described in embryonic development as a process that enables the correct morphogenetic events during migration of epithelial cells from the original position to their ultimate destination. However, EMT also occurs in pathological situations such as cancer (2, 5). During the progression of solid malignancies from benign tumors to locally invading tumors, and finally to metastatic neoplasms, EMT plays a key role. However, it seems that cancer-associated EMT is only activated partially and transiently, in contrast to developmental EMT (3). This attribute and the fact that EMT programs have been associated with other cellular programs such as cell survival, stemness and resistance to drugs (4), makes EMT difficult to study by only analyzing the expression of EMT-TF network.

Recent studies suggest the involvement of EMT in first generation Somatostatin Receptor Ligands (SRLs) resistance in GH-producing pituitary tumors (6–9). Here we review the role of EMT in pituitary adenomas and discuss the relationship between EMT and SRLs resistance in GH-producing pituitary tumors as well as offer new potential biomarkers and therapeutic options.

## Methods

We performed a systematic review with the aim of summarizing the current knowledge of EMT in GH-secreting adenomas with a special focus on SRLs resistance. We performed a search in MEDLINE database using its PubMed tool of the literature available until January 2021. We searched for the terms: “Epithelial-mesenchymal transition” OR “EMT” AND “Acromegaly” OR “Pituitary adenoma” OR “Pituitary tumor” AND/OR “Somatostatin” OR “Somatostatin receptor ligands” OR “Somatostatin analogs”.

## Epithelial–Mesenchymal Transition in Pituitary Tumors

It is well known the importance of the expression of determined transcription factors during pituitary organogenesis to give the final identity to every different hormone-secreting cell type (10), and EMT plays an important role in this embryogenic process (11). The *PROP1* transcription factor, that is vital for the ontogenesis of somatotroph cells, was discovered to promote EMT during pituitary stem cell differentiation, making EMT an important step to obtain fully-functional differentiated pituitary cells (12, 13). These results have been validated by single-cell transcriptomic profiling of the different developmental lineages in human pituitary (14).

EMT is not only linked to pituitary through development, as E-cadherin has been related to hormone secretion in mature cells. E-cadherin reduces prolactin protein content through affecting trafficking of secretory granules (15). Furthermore, it has been also associated with follicle-stimulating hormone (FSH) content and subcellular localization in non-functioning pituitary tumors (16).

EMT also plays an important role in the aggressive biologic behavior of pituitary tumors. Pituitary tumors are the second most common primary brain tumors with invasive properties. The loss of E-cadherin, which is a key characteristic of EMT associated with poor prognosis and high grade tumors in almost all malignancies derived from epithelial cells, has also been reported in pituitary tumors. E-cadherin, a calcium-dependent cell to cell adhesion transmembrane protein, is part of a cell adhesion complex where it is associated with other proteins (α−, β−, γ− and p120-catenins) through an intracellular domain (17). Interestingly, E-cadherin can act as direct transcriptional regulator by nuclear translocation (18). In pituitary tumors, the loss of E-cadherin, specifically the loss of cytoplasmic E-cadherin, is frequently found concurrently with its detection in the nucleus (19). Importantly, nuclear staining E-cadherin is associated with tumor invasion, suggesting that cleavage of the extracellular domain of E-cadherin and nuclear translocation may participate in local invasion in pituitary tumors. Similarly, E-cadherin among other adhesion molecules was related to invasiveness and proliferative status of prolactinomas (20, 21). Other classical EMT markers, such as N-cadherin, *SNAI1*, *SNAI2* and *TWIST* (21, 22) or β-catenin (23) have also been associated with a worse clinical course in pituitary tumors, especially indicating an invasive phenotype, although there is some controversy regarding this subject in acromegaly (21, 24).

Furthermore, different other non-classical molecules related to EMT have been characterized as part of the mechanisms allowing invasiveness in pituitary tumors, such as *ADAM12* (a disintegrin and metalloprotease 12), which has been postulated as an EMT inducer in these tumors (25). *ADAM12* overexpression is associated with pituitary tumor invasiveness, while its silencing prevents such biological behavior. Mechanistically, *ADAM12* silencing impairs ectodomain shedding of epidermal growth factor receptor (EGFR) ligands and attenuated the EGFR/ERK signaling pathway. Inhibition of EGFR signaling resulted in EMT suppression similar to repression of *ADAM12*. Also, a recent study by Falch at al (26). in non-functioning gonadotroph tumors reported that those tumors harboring invasive and rapid growing characteristics showed overexpression of genes involved in EMT, in particular *SPAG9*, *SKIL*, *MTDH*, *HOOK1*, *CNOT6L* and *PRKACB*.

Surprisingly, pituitary tumor transforming gene 1 (*PTTG1*) has been related to EMT in non-functioning pituitary adenomas (27), just the opposite role of *PTTG2* (28). Other authors suggested that the mechanism triggering the EMT in pituitary tumors is linked to the expression of S100A9, a member of the S100 family of EF-hand motif Ca^2+^-binding proteins, mediated by activation of AKT1 (29). In addition, it has been showed that the transcriptoma of *USP8* wild-type corticotropinomas, characterized by increased invasiveness, was enriched in EMT signature (30).

In another study in GH-secreting adenomas, cyclin B1 (*CCNB1*) knock-down was found to decrease the mesenchymal marker N-cadherin and increase the epithelial markers E-cadherin and p120-catenin. Thus, inactivation of cyclin B1 results in a decreased proliferation and EMT, and an increased apoptosis (31). A similar approach was used in a different study where the expression of *SMAD4* was found to regulate EMT in somatotropinomas. *SMAD4* was associated with invasion, increased levels of vimentin and N-cadherin and, decreased E-cadherin (32).

Other genes have been related to the suppression of EMT and, therefore, invasion. It is the case of collagen type VI alpha 6 (*COL6A6*) that inhibits cell proliferation, migration, invasion, and epithelial-mesenchymal transition (EMT) through the binding of *P4HA3* resulting in PI3K-Akt axis inhibition in pituitary adenomas (33).

Not only coding genes have been related to EMT in pituitary tumors, some microRNAs (miRNAs) and long non-coding RNAs (lncRNAs) have been shown to modulate EMT. MiR-149-5p and miR-99a-3p suppress the expression of EMT-related genes (34). miR-132, miR-15a and miR-16 also inhibit EMT in pituitary adenomas; in this case targeting *SOX5* (35). Moreover, miR-424-3p inhibits EMT and invasion by targeting *JAG1* (36). On the other hand, lncRNAs seem to be related to EMT enhancement rather than inhibition. For example, lncRNA *SNHG6* induces EMT suppressing miR-944, which may inhibit *RAB11A* (37). Furthermore, lncRNA *PVT1* enhances EMT and migration by activating Wnt/ß-catenin (38). Finally, lncRNA *SNHG1* promotes EMT and invasion by activation of *TGFBR2*/*SMAD3* and *RAB11A*/Wnt/β-Catenin axis, and the inhibition of miRNAs such as miR-302/372/373/520 (39).

EMT is a dynamic process, not a binary process, with intermediary states (2). It is very unlikely that benign tumor cells undergo a complete mesenchymal transformation which is associated with metastatic tumors (5). Because of that, it is more likely that, as in the majority of neoplasms, pituitary tumors would exhibit partial EMT states (40). This would explain why in transcriptomic analysis some EMT markers are up-regulated while others do not, instead of showing a complete mesenchymal profile (9, 41).

It is noteworthy to highlight the importance of the tumor microenvironment in mediating EMT and, therefore, the aggressive behavior of pituitary adenomas. The alteration of the tumor microenvironment seems to be triggered by tumor chemokines that attract immune cells (42). Additionally, IL-6 and CCL2 produced by tumor associated fibroblasts have been associated with EMT-like morphological changes and aggressive behavior trough E-cadherin downregulation and *ZEB1* upregulation in an *in vitro* study (43).

It is really important to confirm the link between EMT and *AIP*, since *AIP*-*ZAC1* pathway is one of the main molecular mechanisms described for SRLs resistance (44). SRLs can activate *AIP* which inhibits adenylate cyclase, reducing cyclic AMP levels. On the other hand, *AIP* activates *ZAC1*. This molecule binds directly p53 and activates gene transcription; moreover, p53 arrests cell cycle, through p21 interaction, and increases apoptosis (45). Deeper explanation of the pathway could be found in some other reviews (45, 46).

## E-Cadherin Loss Is an Outstanding Biomarker for SRL Resistance in Acromegaly

Somatostatin is secreted by the hypothalamus and inhibits hormone secretion and to a lesser extent pituitary cell growth by binding to different G protein-coupled receptors (SSTR1–5) [reviewed in Ben-Shlomo and Melmed (47)]. As remnant of its somatroph origin, somatotropinomas express somatostatin receptors, specially *SSTR2* and *SSTR5* (48). First-generation Somatostatin Receptor Ligands (SRLs), octreotide and lanreotide, so far the accepted first-line medical therapy in acromegaly despite that hormonal hypersecretion control of the disease is generally reported to be lower than 50% and both show a high affinity for *SSTR2* receptor (49). Molecular characterization of the tumors has unveiled several explanations for such uneven response (50). However, many studies have proved the involvement of many other players and nowadays we do not know the whole picture of SRLs resistance in acromegaly.

EMT and epithelial plasticity have been associated with resistance to conventional, targeted and immune therapies in many cellular and preclinical models in different tumor contexts, although there is little evidence from clinical samples (51). In this line, different studies relate EMT and SRLs resistance in acromegaly. E-cadherin has been linked to SRLs response as an independent predictor by different studies and in different cohorts (8, 52, 53). In a fair comparison between several known biomarkers of SRLs response, E-cadherin showed the greatest performance in predicting postsurgical SRLs response, even greater than *SSTR2* or Ki-67 (8). There is a general consensus that low levels of E-cadherin mRNA and protein indicate a poor responsive tumor to SRLs (8, 52, 53). Furthermore, E-cadherin loss seems to be related to the granulation pattern of the tumor, especially but not exclusively in GH-producing tumors (8, 53). It is worth saying that the histological granulation pattern of the tumor has been related to SRLs response for many years (54, 55). Interestingly, some studies have shown the association between E-cadherin downregulation and E-cadherin promoter hypermethylation in GH-secreting tumors, suggesting the involvement of epigenetic mechanisms (56–58). Another study pointed to the presence of progenitor mesenchymal cells derived from cancer stem cells as the cause of E-cadherin decrease and EMT induction (through TGFbRII increase) in somatotropinomas (59).

Taking into account that E-cadherin is routinely assessed in pathology departments as diagnostic tool for other cancer types (60), it would be easy to implement it as biomarker of response to SRLs to better define acromegaly treatment (61).

## Beyond E-Cadherin Loss: Involvement of Other Epithelial–Mesenchymal Transition Molecules in SRL Resistance in Acromegaly

Since E-cadherin loss is a marker of advanced EMT, some authors have further investigated this phenomena in GH-producing tumors. Lekva and colleagues analyzed the transcriptome of tumors with very high and very low levels of E-cadherin and identified several EMT-related genes. Interestingly, *in vitro*, the expression of these genes were not regulated by E-cadherin but by Epithelial Splicing Regulatory Protein1 (*ESRP1*) (6). *ESRP1* has been characterized as an important contributor to EMT by mediating alternative splicing in EMT affecting the maintenance of epithelial features (62). It is important to mention that several studies have proved the relation of splicing and SRLs resistance in acromegaly (7, 63–65). *ESRP1*, thus, may be a master regulator of the EMT, SRLs response and other pathological processes in acromegaly (7).

Lekva and colleagues also investigated genes that were differentially expressed upon treatment with SRLs in different EMT contexts. They found that RAR Related Orphan Receptor C (*RORC*) was overexpressed in phenotypically epithelial tumors but not in mesenchymal ones (9). Moreover, *RORC* expression was associated with SRLs response, a result that has been confirmed by finding that *RORC* is a biomarker of SRLs response improvement after surgical debulking (66).

On the other hand, patients harboring *AIP*-mutated somatotropinomas tend to be diagnosed at a younger age with larger, more aggressive, and SRLs resistance tumors (44). Some studies have shown that *AIP* is an important mediator of SRLs response (45), and *AIP* expression has been found to be a potent SRLs response predictor (44). In this context, it was interesting to prove that the transcriptome of ten somatotropinomas and five normal pituitaries revealed EMT as one of the most significantly altered pathways in *AIP*-mutated tumors. Furthermore, the cell-conditioned media of AIP-knockdown cells increases migration of macrophages (41), reinforcing the role of tumor microenvironment in inducing EMT and a more aggressive phenotype.

## Cytoskeleton, Epithelial–Mesenchymal Transition, and SRL Resistance in Acromegaly

One of the main characteristics of EMT is the reorganization of cell polarity through changes in the cytoskeleton, which is composed of the actin cytoskeleton, the microtubule network and the intermediate filaments that provide structural design and mechanical strength. The cytoskeleton is known to play an important role in EMT during cancer progression (67). Concretely, refilin proteins perform their function through filamin A (*FLNA*) to regulate the actin cytoskeleton reorganization. RefilinA promotes the conversion of *FLNA* from an actin branching protein into an F-actin bundler, and RefilinB combined with *FLNA* organize a unique perinuclear actin network at the apical surface during the EMT (68, 69). Interestingly, in *SNAI1*-induced EMT, it has been proved that the changes in nuclear morphology and in the cytoskeleton structure correlate with decreased expression of *FLNA* (70).

*FLNA* plays an important role in GH-producing tumors since it has been related to pituitary tumors migration and invasion (71) and, more importantly, also to SRLs resistance (72). Additionally, it has been proved that *FLNA* mediates octreotide-induced *SSTR2* trafficking through endosomal proteins in acromegaly. Moreover, *FLNA* influences the number of available *SSTR2* at the surface of the cell (73). For more detailed explanation of the cytoskeleton involvement in SRLs resistance, we recommend the review by Peverelli et al. published in 2015 in this same journal (74).

## Epithelial–Mesenchymal Transition-Related Therapies

The involvement of EMT in acromegaly pathogenesis and SRLs resistance offers new therapeutic approaches that should be explored. As an example, *CCNB1* overexpression in acromegaly can be targeted with resveratrol, inhibiting *CCNB1* and reverting its effects on invasion (31). Interestingly, Pasireotide, a second generation SRL, has been associated with a reduction of EMT-associated chemokines in tumor associated fibroblasts, suggesting an anti-tumor effect targeting the microenvironment (43). In contrast, other first generation SRLs do not appear to affect EMT (59).

Several EMT regulating TFs (*SNAI1*, *SNAI2*, *TWIST*…) can induce a therapy-resistant intrinsic mechanism (overexpression of drug efflux pumps) as well as an extrinsic one (gaining resistance to apoptosis inducing agents). This explains why EMT is often related to drug resistance in tumors (2). However, EMT features are emerging as novel therapeutic targets in cases of resistance to current therapies (75). Some of the drugs proposed to inhibit EMT in clinical phases are well-known for endocrinologists such as metformin (76). Others have been proposed to be useful in acromegaly to target GDNF-RET/PIT1/p14ARF/p53 pathway, like Sorafenib (77).

EMT offers target opportunities in different levels: inhibiting stimuli from the tumor microenvironment, inhibiting extracellular mediators and their corresponding receptors, inhibiting or activating intracellular signaling pathways, and inhibiting transcription factors that indirectly induce EMT (78). On this last regard, the usage of an inhibitor of *STAT3* could very much benefit acromegaly therapy since it would act reverting EMT process (79–81) and directly inhibiting GH hypersecretion (82). More than a dozen of different therapies targeting EMT are being tested in clinical trials, however the vast majority are used in combination with regular chemotherapy since it is expected to recover sensitivity of more conventional drugs upon EMT inhibition (78). In acromegaly, the main concern rather than the proliferation and formation of metastasis is the normalization of hormone levels; this is the reason why rather than the expected antiproliferative effects of these drugs, we would expect a resensitization to SRLs. However, nowadays it is unknown if this effect would be achieved and which molecules should be targeted.

## Discussion

It is very likely that EMT plays an important role in acromegaly pathogenesis, but also in the modulation of pharmacologic response, thus inducing SRLs resistance in particular. However, most of this relationship is unknown since the molecular pathways relating EMT and SRLs signaling are not really understood and sufficiently explored. We are only beginning to unveil this relationship and we have, by now, been able to find some of the key molecules, but the whole picture remains elusive. To add a little more complexity to the acromegaly and EMT relationship, it is worth to mention that one of the surprising effects of GH is the induction of EMT (83), closing the circle around EMT and acromegaly. The induction of EMT by GH seems to be mediated by the tumor microenvironment involving not only tumor cells but multiple non-tumoral cell types (84, 85).

Importantly, the study of EMT has provided some interesting biomarkers to predict SRLs response in acromegaly, for example, E-cadherin and *RORC*. Furthermore, as we could only glimpse for now, EMT in acromegaly is involved in a lot of processes like stemness, apoptosis, secretory vesicles trafficking, cytoskeleton organization, invasion capacities and aberrant splicing. All of them are, to some extent, individually related to SRLs resistance and that makes very difficult to delimitate the action of EMT. It forces to contemplate EMT as a dynamic process with deep connections with a multitude of different cellular programs. Moreover, the presence of intermediate EMT states in tumors, which generates tumor heterogeneity, is probably key in the contribution of EMT to treatment resistance.

Further studies of the EMT process would not only provide in-deep knowledge about the dedifferentiation of GH-secreting tumors and the SRLs desensitization, but will certainly offer alternative treatments to the SRLs. Several EMT inhibitors are currently been tested in clinical trials for other malignancies. The plastic and dynamic nature of EMT increases the difficulty in determining the appropriate therapeutic and diagnostic windows. However, targeting EMT blockade as an adjuvant therapy could potentially increase the effectiveness of the GH-secreting tumors to SRLs.

In conclusion, EMT is a process that plays an important role in the heterogeneity of pituitary adenomas and is associated with a more aggressive phenotype. Furthermore, it has been linked to SRLs response in somatotropinomas. Thus, EMT-related therapies may be taken into consideration in the treatment of acromegaly, especially in SRLs non-responder patients. This could be an opportunity to find new therapies for pituitary adenomas; however, the increasing therapeutic options for acromegaly may overwhelm clinicians making more difficult the choice of the best molecule(s) to target. In this regard, some authors have developed a universal and quantitative EMT scoring based on transcriptomic data that allows the prediction of response to different pharmacological treatments (86). We think that this type of tools should be the basis of the future medicine in acromegaly; the “trial‐and‐error” approach to decide the appropriate drug would no longer be an option (87–90).

## Author Contributions

All authors listed have made a substantial, direct, and intellectual contribution to the work and approved it for publication.

## Funding

Work in our laboratory is supported by the Instituto de salud Carlos III (PM 15/00027) and Novartis through the REMAH (Registro Español Molecular de Adenomas Hipofisarios) consortium to the SEEN (Sociedad Española de Endocrinología y Nutrición).

## Conflict of Interest

MP-D declares to have received funding from Novartis through the REMAH consortium for research purposes.

The remaining authors declare that the research was conducted in the absence of any commercial or financial relationships that could be construed as a potential conflict of interest.
